# Proteomic analysis of dimorphic sperm in the cabbage white butterfly, *Pieris rapae*

**DOI:** 10.3389/finsc.2026.1772436

**Published:** 2026-03-25

**Authors:** Melissa Plakke, Katherine McLaughlin, Timothy L. Karr, James R. Walters

**Affiliations:** 1 Division of Science, Mathematics, and Technology, Governors State University, University Park, IL, United States; 2 Department of Ecology and Evolutionary Biology, University of Kansas, Lawrence, KS, United States; 3 Biosciences Mass Spectrometry Core Research Facility, Knowledge Enterprise, Arizona State University, Tempe, AZ, United States; 4 ASU-Banner Neurodegenerative Disease Research Center, Arizona State University, Tempe, AZ, United States

**Keywords:** butterfly, lepidoptera, mass spectrometry, proteomics, sperm

## Abstract

Sperm dimorphism, the production of two distinct sperm morphs by a single male, is a widespread but enigmatic reproductive phenomenon. In Lepidoptera, fertilizing eupyrene sperm coexist with anucleate apyrene sperm, which cannot fertilize eggs but are nevertheless required for successful reproduction. Despite the prevalence and presumed adaptive significance of sperm dimorphism, the molecular basis of this trait remains limited. Here, we characterize the proteome of dimorphic sperm in the Cabbage White butterfly, *Pieris rapae*, an emerging model for sexual selection and postcopulatory interactions. Using high-resolution, label-free mass spectrometry, we identified more than 1,600 proteins, nearly doubling the number of proteins previously reported for other lepidopteran species. Differential abundance analyses revealed eupyrene sperm were enriched for proteins linked to ion transport and vacuolar acidification, while apyrene sperm were enriched for mitochondrial and respiratory functions. Unexpectedly, comparative homology analyses with two other Lepidoptera, *Danaus plexippus* and *Manduca sexta*, showed *P. rapae* shared more homologous sperm proteins with *M. sexta* than with the more closely related *D. plexippus*, highlighting complex evolutionary dynamics of sperm proteomes. Together, these findings expand our understanding of sperm function and diversity in Lepidoptera, highlight the distinct roles of eupyrene and apyrene sperm, and provide a foundation for future studies of sperm evolution, sexual selection, and reproductive protein function.

## 1 Introduction

In sexually reproducing species, the gametes play a pivotal role in determining fitness. Although sperm’s primary role is to contribute genetic material during the process of fertilization, sperm cells exhibit significant diversity across various organisms in relation to movement, shape, and size (1, 2). For instance, in mice, sperm cells attach to each other and form “sperm trains” for increased swimming speed (3) while in nematodes sperm moves in an amoeba-like fashion (4). Songbirds exhibit helically-shaped sperm heads (5) and *Drosophila bifurca* is famous for its sperm gigantism (6).

One of the most striking examples of sperm diversity is sperm dimorphism. Sperm dimorphism consists of a single male producing two distinct sperm types. This phenomenon has arisen independently multiple times across lineages, including mollusks, echinoids, fish, and insects (6–11). In numerous instances of dimorphic sperm, one type of sperm acts as the fertilizing sperm (the eusperm) while the other type is a non-fertilizing sperm (the parasperm). The repeated evolution of dimorphic sperm indicates its importance in evolution as a presumably adaptive trait, though the purpose remains largely unknown. There are several hypotheses surrounding the function of parasperm, the most prominent being: (1) to mediate sperm competition, (2) to aid in eusperm fertilization, and (3) to provide nuptial gifts and nutrients for the female and developing eggs (10, 12).

Lepidoptera not only possess dimorphic sperm but also present an extreme version of this trait. In Lepidoptera, the fertilizing eusperm are called “eupyrene”, and appear morphologically similar to sperm in other insect groups (13). Lepidopteran parasperm, termed “apyrene”, lack a nucleus and nuclear DNA; they do not participate directly in the process of fertilization (13). Despite lacking genetic material and the ability to fertilize eggs, apyrene sperm are necessary for successful fertilization (14–16).

Recent advances in genomic-scale analyses have created novel opportunities to assess the functional, evolutionary, and molecular characteristics of dimorphic sperm. In particular, mass spectrometry-based proteomics are especially helpful in the identification of reproductive proteins (17) and offer a direct link between genotype and phenotype (18). Such proteomic approaches have proven highly successful in directly assaying the protein contents of sperm and can also reveal evolutionary insights. For instance, previous proteomic analysis of dimorphic sperm in Lepidoptera uncovered variation in the evolutionary dynamics of protein composition between the morphs. In comparisons between *Danaus plexippus* (monarch butterfly) and *Manduca sexta* (hornworm moth), the proteins that are found in both sperm morphs were relatively more conserved across species than morph-specific proteins. This elevated rate of turnover is most striking in apyrene sperm, as the apyrene sperm demonstrate the highest level of unique proteins when compared across species (19). Subsequent molecular evolutionary analyses of these dimorphic sperm proteomes indicated that sperm competition appears to have a stronger impact on eupyrene than apyrene sperm (20).

These previous studies from monarch and hornworm revealed the variability of lepidopteran sperm proteomes and underscore the need for expanded research characterizing the molecular and functional diversity of dimorphic sperm. Such work is ideally conducted in systems with prior complementary studies characterizing proteins within both the male and female reproductive tracts*. Pieris rapae*, the cabbage white butterfly, is an emerging model for understanding the different dynamics of male-female interactions during and after mating (21–25). Several studies demonstrate the manipulation of apyrene versus eupyrene allocation in relation to sperm competition. For example, *P. rapae* are shown to tailor the ratio of sperm morphs within a single ejaculation event in response to a female’s mating history, increasing the overall volume and the relative amount of apyrene sperm transferred if a female has been previously mated (12). Recent proteomic and transcriptomic work has focused on the non-sperm proteins present in the spermatophores and their interactions with the female bursa copulatrix (26). These studies’ results suggest a link between sexual conflict and reproductive trait evolution at the interface between male sperm competition and female control of remating efforts (26).

Given the growing precedent of molecular and proteomic studies of reproductive processes, male-female interactions, and sperm competition in *P. rapae*, we sought to characterize the proteome of apyrene and eupyrene sperm in this system. With the rapidly advancing technologies available for discovery-based, label-free proteomics, we aim to catalog proteins present within each morph as well as determine general proteins shared between the morphs. Further, by adding a third lepidopteran species to our growing database of sperm proteomes, we will be able to establish clearer lines between derived and ancestral traits.

## 2 Methods

### 2.1 Sample collection

**Figure 1:**
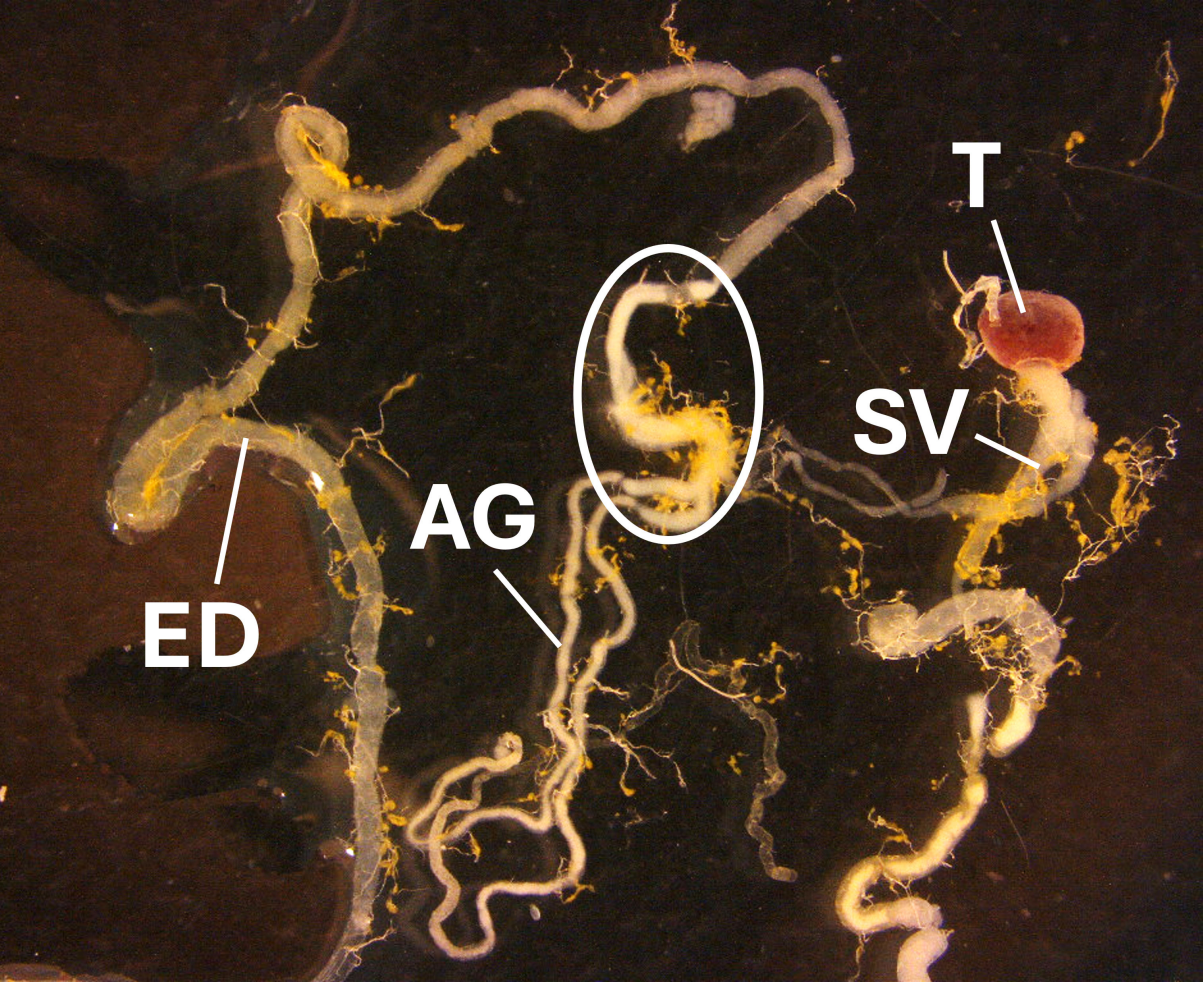
*Reproductive tract of a male Pieris rapae.* Testes (T) release mature sperm of both morphs into the paired seminal vesicles (SV). Sperm migrates and is stored until mating past the junction with the accessory glands (AG) at the transition between the duplex and simplex region of the ejaculatory duct (ED). Sperm for proteomic analyses were extracted from this region, circled in the image.

*Pieris rapae rapae* (Linnaeus 1758) individuals sampled in this study were caught from wild populations of unknown mating status in the vicinity of Lawrence, KS during the summer of 2020. All dissections and subsequent sperm panning were conducted in phosphate-buffered saline (PBS) solution mixed with 1x HALT protease inhibitor (Thermo Fisher Scientific). Abdomens of insects were cleared of scales using cellophane tape prior to dissection to prevent contamination. Adult males were vivisected and sperm were released from the simplex-duplex junction of the reproductive tract (**Figure 1**). The freed sperm were collected via pipette and transferred to a separate, smaller petri dish (60mm x 15mm) filled halfway with PBS-protease inhibitor solution.

For each butterfly, the sperm were separated into their respective eupyrene and apyrene components using the sperm panning method (27). Described briefly, this method employs orbital agitation to consolidate eupyrene bundles in the center of the dish while individual apyrene sperm dissipate to the dish periphery. Separated fractions of apyrene or eupyrene sperm were pooled for three males to generate a single biological replicate for each sperm morph. The eupyrene sperm isolated by panning were transferred in ~300 µL to a single 1.5 mL Eppendorf tube and pelleted by centrifugation at 14000 rpm for 15 minutes. To collect and consolidate apyrene sperm from the periphery of the dishes, two mL per butterfly were evenly distributed into six Eppendorf tubes and centrifuged for 15 minutes at 14000 rpm to pellet the sperm. Subsequently, the sperm pellet in 100 *μL* of supernatant from each tube was combined to one Eppendorf tube and again pelleted by centrifugation for 15 minutes at 14000 rpm, forming a single biological replicate. The resulting samples of separated apyrene and eupyrene sperm were then frozen at -80 C until submission for mass-spectrometry analysis. Due to variation in the panning and sampling process, the biological replicates for eupyrene and apyrene morphs sent for proteomic analysis are not properly paired and cannot be analyzed as paired samples.

### 2.2 Mass spectrometry proteomic analysis

A total of 12 biological replicates were analyzed, six each for eupyrene and apyrene. Five biological replicates of each sperm type were analyzed in triplicate technical replicates but the last biological sample for each sperm type was analyzed in duplicate only, due to technical constraints of the mass spectrometer relative to the number of samples provided. Thus, data from a total of 34 technical replicates were obtained.

Replicates were analyzed via mass spectrometer at the Biosciences Mass Spectrometry Core Facility (https://cores.research.asu.edu/mass-spec/) at the Arizona State University. Data-dependent mass spectra were collected in positive mode using an Orbitrap Fusion Lumos mass spectrometer (Thermo Scientific) coupled with an UltiMate 3000 UHPLC (Thermo Scientific). 1 µL of peptides were fractionated using an Easy-Spray LC column (50 cm × 75 µm ID, PepMap C18, 2 µm particles, 100 Å pore size, Thermo Scientific) equipped with an upstream 300um x 5mm trap column. Electrospray potential was set to 1.6 kV and the ion transfer tube temperature to 300°C. The mass spectra were collected using the “Universal” method optimized for peptide analysis provided by Thermo Scientific. Full MS scans (375–1500 m/z range) were acquired in profile mode with the Orbitrap set to a resolution of 120,000 (at 200 m/z), cycle time set to 3 seconds and mass range set to “Normal”. The RF lens was set to 30% and the AGC set to “Standard”. Maximum ion accumulation time was set to “Auto”. Monoisotopic peak determination (MIPS) was set to “peptide” and included charge states 2-7. Dynamic exclusion was set to 60s with a mass tolerance of 10ppm and the intensity threshold set to 5.0e3. MS/MS spectra were acquired in a centroid mode using quadrupole isolation window set to 1.6 (m/z). Collision-induced fragmentation (CID) energy was set to 35% with an activation time of 10 milliseconds. Peptides were eluted during a 240-minute gradient at a flow rate of 0.250 uL/min containing 2-80% acetonitrile/water as follows: 0–3 minutes at 2%, 3–50 minutes 2-15%, 50–120 minutes at 15-30%, 120–160 minutes at 30-35%, 160–170 minutes at 35-80% and 170–180 at 80%. Each run was followed by a 30-minute wash using 2-90% acetonitrile/water.

### 2.3 Database searching and label-free quantification

As a database for peptide matches, we used a curated version of the *P. rapae* predicted protein set (28). In this resource, many genes were annotated with multiple transcripts that were identical or encoded identical proteins. We therefore removed such redundant protein annotations by randomly selecting one transcript among each set of transcripts producing identical proteins. Thus, all distinct protein isoforms per gene were represented in our database, but each protein was unique.

Raw files were searched against this curated *P. rapae* protein database using Proteome Discover 2.4 (Thermo Scientific). Raw files were searched using SequestHT that included Trypsin as enzyme, maximum missed cleavage site 3, min/max peptide length 6/144, precursor ion (MS1) mass tolerance set to 20 ppm, fragment mass tolerance set to 0.5 Da and a minimum of 1 peptide identified. Carbamidomethyl (C) was specified as fixed modification, and dynamic modifications set to Aceytl and Met-loss at the N-terminus, and oxidation of Met. A concatenated target/decoy strategy and a false-discovery rate (FDR) set to 1.0% was calculated using Percolator (1). The data was imported into Proteome Discoverer 2.4, and accurate mass and retention time of detected ions (features) using Minora Feature Detector algorithm. The identified Minora features were then used to determine area-under-the-curve (AUC) of the selected ion chromatograms of the aligned features across all runs and relative abundances calculated.

### 2.4 Differential abundance analysis

Qualitative detection (*i.e.*, protein presence and absence) and label-free quantification (*i.e.*, normalized precursor ion abundances) obtained from Proteome Discoverer were used as input for subsequent differential abundance analyses. Statistical analysis and related visualizations were completed with custom scripting in R, relying heavily on the DEP package (29). The DEP package provides a robust framework for statistical testing of differential abundance in proteomic data, but does have a limitation: the package is not configured to analyze technical replicates nested within biological replicates. Therefore, abundance values were averaged across technical replicates to obtain an abundance value for each biological replicate. Very high correlations between technical replicates indicated that averaging them was acceptable (see Results for details). The resulting abundance values among the biological replicates were used as input for analysis using functions implemented in DEP. Accordingly, all statistical inferences were performed at the level of biological replicates; technical replicates were employed only to increase the accuracy of protein abundance estimation from biological replicates.

### 2.5 Filtering

Initial inspection of the data indicated one apyrene biological replicate had very low counts of detected proteins and an unusually high distribution of abundances, so we removed this replicate from the analysis (see Results for details). Subsequently, data were filtered to remove sporadically detected proteins. Filtering was applied based on the number of biological replicates in which a protein was detected. Proteins were removed that had greater than two missing observations in both sperm morphs.

### 2.6 Imputation

After filtering, any proteins with missing values were classified as having values either *missing at random* (MAR) or *missing not at random* (MNAR). Two or more observations were required in both morphs to be MAR. MNAR had zero or one observation(s) in one sperm morph type, and at least three in the other. A mixed approach was used for imputation when analyzing MNAR and MAR protein detection. The “MinDet” method was used for MNAR and “MLE” was used for MAR, as implemented in the DEP package.

### 2.7 Assessing significance

The DEP package employs the linear modeling framework implemented in R package limma (30) to assess differentially abundant proteins between samples. Using DEP, we implemented a pairwise contrast between apyrene versus eupyrene sperm samples, using Benjamini-Hochberg (BH) false-discovery rate corrected p-values <0.05 to determine statistical significance (31). We further required a 1.5-fold difference in average abundance between sperm morphs for a protein to be considered differentially abundant.

### 2.8 Qualitative assessment

In addition to statistical assessment of differential abundance using imputed data, a qualitative assessment was conducted on the data prior to imputation to safeguard against DEP imputing values too conservatively. To be considered qualitatively differentiated between apyrene and eupyrene sperm, proteins were required to be present in at least four biological replicates of one sperm morph type and present zero or one time(s) in the other sperm morph type. Permitting one replicate allows for some error due to contamination during sperm separation.

### 2.9 Functional analysis between sperm morphs

Sperm protein sequences were functionally annotated using a range of bioinformatic approaches. When multiple isoforms were present for a given gene, the longest protein isoform was selected to represent the gene for bioinformatic annotations. Proteins were analyzed via BLASTp against the SwissProt database and the top hit was recorded (32). Structural motifs corresponding to signal peptides (indicating secretion), targeting to the mitochondria, or transmembrane domains were inferred, respectively, using SigalP (v6), TargetP (v2), and TMHMM (v2) softwares (33–35). The proteins were also analyzed via the PANNZER software to get a descriptive functional prediction and gene ontology category assignments (36). GOStats was used for gene ontology enrichment analysis (37). Enrichment tests were conducted separately for the three categories of proteins based on differential abundance: eupyrene-biased, apyrene-baised, or unbiased. Each of these subsets was compared to the background set of all detected sperm proteins using the conditional version of the hypergeometric test implemented in GOstats.

### 2.10 Homology analysis across species

Gene orthologs were predicted between *P. rapae*, *M. sexta*, and *D. plexippus* using the ProteinOrtho pipeline, using the longest isoform for each gene as the representative protein in each species (38). Sperm proteins identified in the mass spectrometry analysis were cross-referenced with those found in *M. sexta* and *D. plexippus* to determine sperm homology (19). For each species, when multiple isoforms were present for a given gene, the longest protein isoform was selected to represent the gene for ortholog predictions.

## 3 Results

### 3.1 Separation of sperm morphs by panning

As a qualitative assessment for the efficiency of separation via the panning method, aliquots from representative samples of separated apyrene and eupyrene sperm were inspected under a microscope (**Supplementary Figure S1**). We observed very strong enrichment for each sperm morph, but not perfect isolation. Some apyrene sperm were visible in the eupyrene sample, and occasional eupyrene bundles were observed in the apyrene samples. While not formally quantified, we roughly estimated that the panning produced a 100- to 1000-fold enrichment for each sperm morph. Accordingly, because the panning method did not achieve complete isolation, we subsequently frame our results for differential protein abundance as being biased towards, but not specific to, the two sperm morphs.

### 3.2 Protein identification, abundance, and filtering

Initially, the results from Proteome Discoverer yielded 1,814 distinct proteins identified that corresponded to 1,796 genes. A comprehensive set of results is provided in **Supplementary Table 1**, including identified proteins, differential abundance estimates, functional annotations, and orthology predictions, as discussed in the following results sections. To streamline downstream quantitative abundance analysis (e.g. the DEP software package does not support statistical models incorporating technical replicates), technical replicates were averaged (mean) to obtain one representative value for each protein for each biological replicate (simply “replicate” hereafter). This decision to use the mean value was supported by the very high consistency between technical replicates; within biological replicates all pairwise correlations between technical replicates were greater than 0.9946.

**Figure 2:**
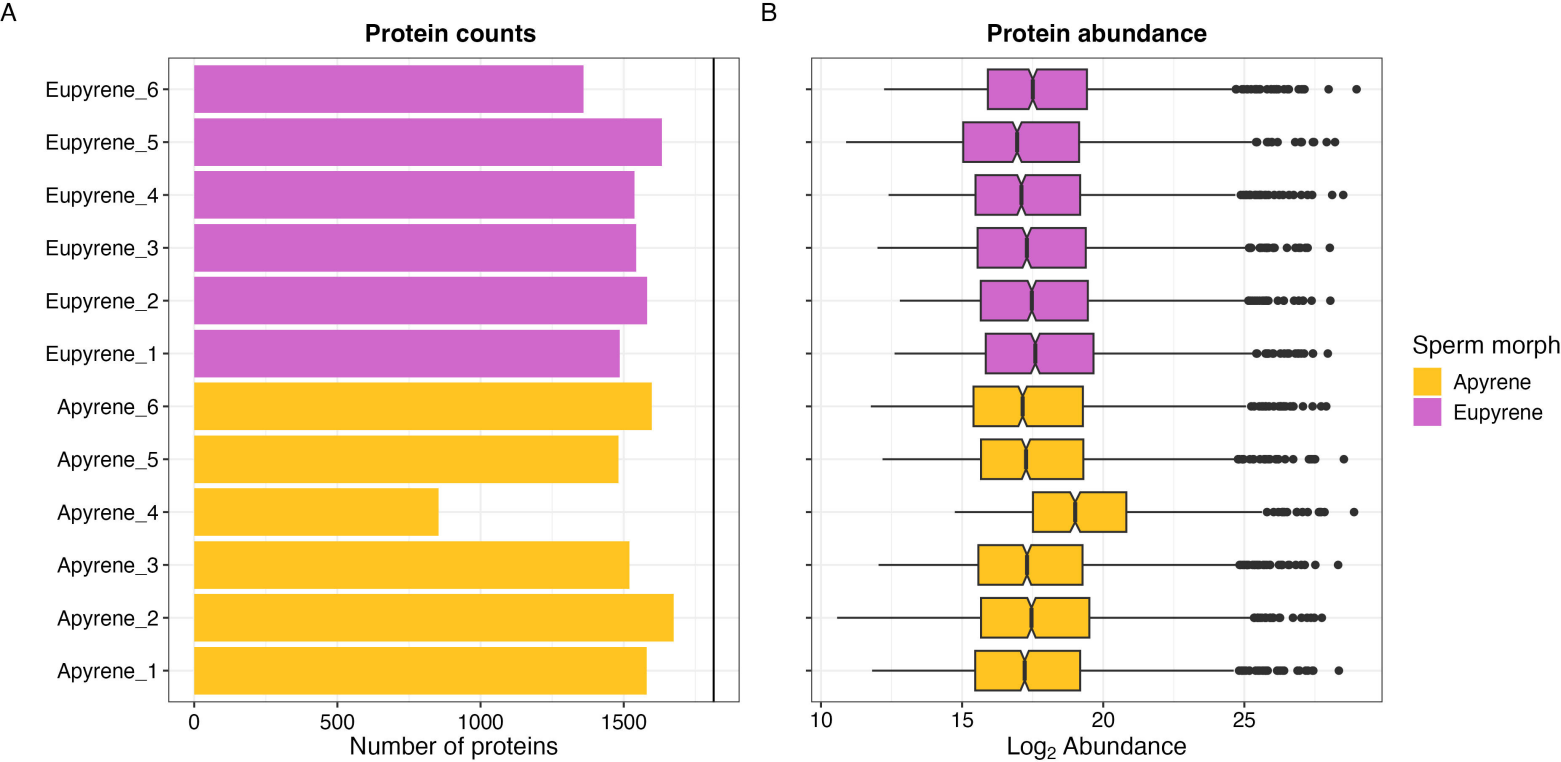
*Mass Spectrometry outputs by biological sample.* Counts **(A)** and abundances **(B)** of proteins observed in each biological sample of eupyrene (purple) or apyrene (gold) sperm prior to data filtering. The vertical black line in the plot of counts represents the total number of unique proteins detected across all replicates prior to filtering. Note that eupyrene and apyrene samples are not paired; having the same identifier numbers does not indicate they come from the same individuals.

Preliminary comparisons of replicates for protein counts and abundances revealed one obvious outlier replicate: sample Apyrene 4. This sample had about half the number of proteins detected relative to all other samples, as well as a substantially elevated distribution of abundances (**Figure 2**). As we could not readily explain this exceptional pattern, we assumed it reflected some unknown analytical or handling artifact, thus we excluded Apyrene 4 from subsequent analyses.

For the remaining 11 replicates, we filtered out inconsistently detected proteins, removing proteins without observations in three or more replicates in both apyrene and eupyrene replicates. This resulted 1,662 consistently detected proteins corresponding to 1,647 genes across all 11 replicates. The majority of these proteins (~1100) were detected in all 11 samples. This pattern holds true for each sperm morph as well (**Supplementary Figure S2**).

**Figure 3:**
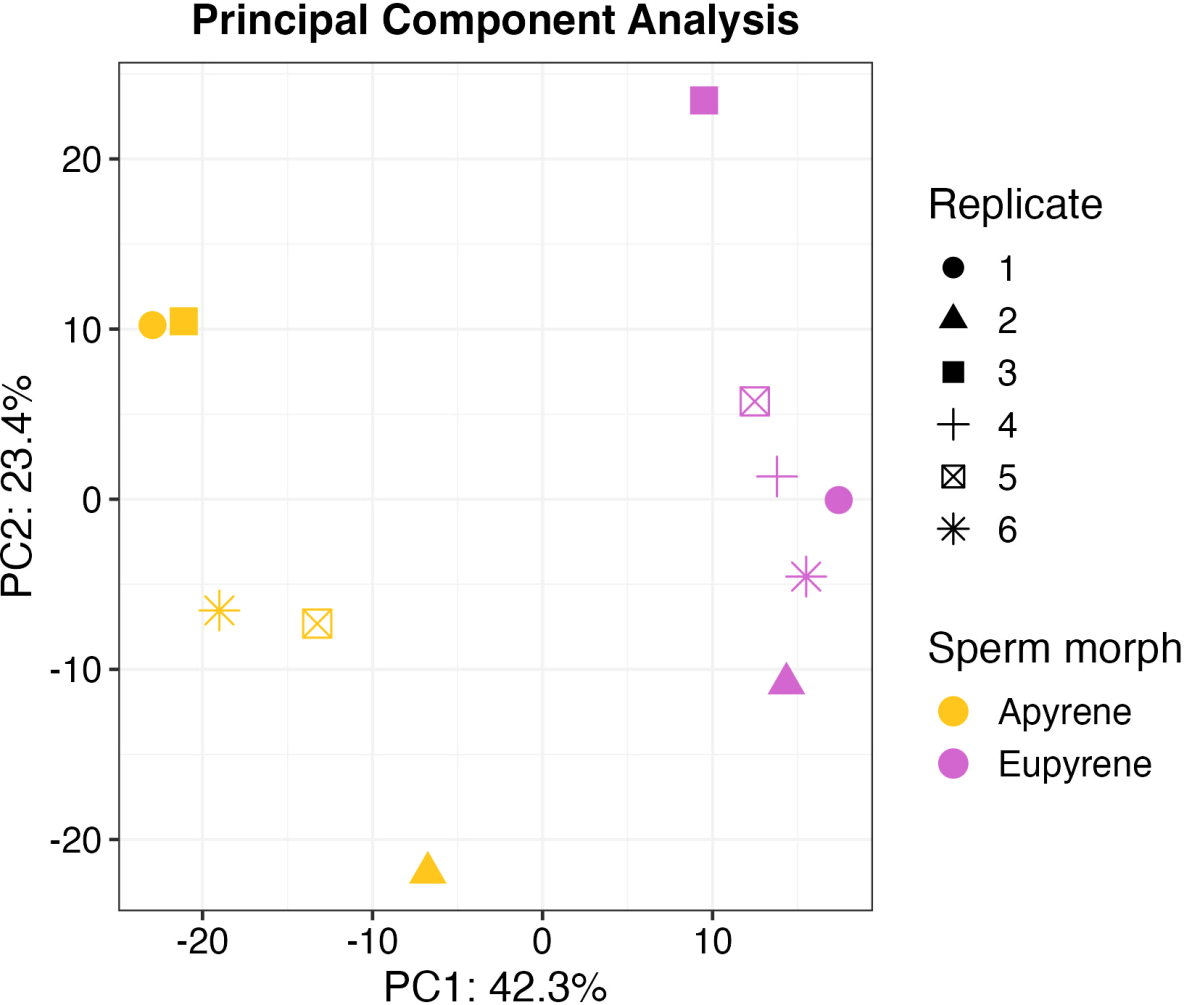
*Principal Component Analysis of* sp*erm morph samples.* PCA using ion intensity abundance values for proteins collected from 11 sperm samples (5 apyrene and 6 eupyrene) distributes the samples by morph type along PC1 (42.3%). PC2 explains 23.4% of the variation between samples.

After filtering, 565 identified proteins had missing observations in at least one biological replicate. Such missing data can be problematic for differential abundance analysis, prompting the imputation of missing values (39). For each protein, missing observations were categorized as either missing at random (MAR) or missing not at random (MNAR). 510 proteins were classified MAR, assuming missing observations arise from stochasticity in detection. 55 proteins were classified MNAR, assuming missing observations reflect qualitative biological differences (**Supplementary Figure S3**). After imputation reflecting this classification, we applied principal component analysis to characterize overall patterns of variation in the data (**Figure 3**). We found that PC1 primarily differentiates replicates corresponded to sperm type and explained ~42% of the variance among replicates.

### 3.3 Differential abundance analysis

We used two approaches for characterizing differential abundance: (1) quantitative statistical methods implemented in DEP and (2) qualitative assessment of presence-absence patterns on non-imputed data. 240 proteins were found to be differentially abundant via DEP and 41 proteins were found to be qualitatively differentially abundant. Only six proteins assessed as qualitatively different were also detected as quantitatively different in DEP using the imputed data.

**Figure 4:**
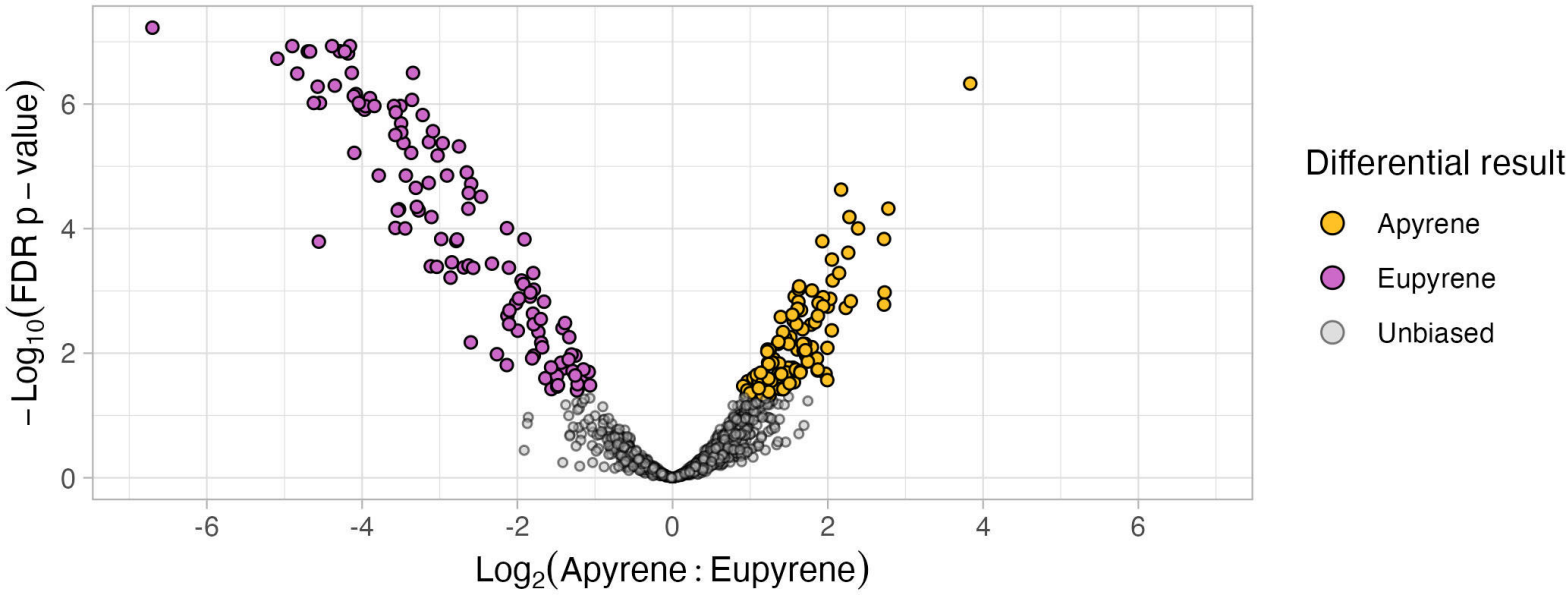
*Differential abundances of proteins between* sp*erm morphs.* Differences in abundance for each protein between each morph are plotted in relation to the overall abundance of each protein. Proteins are colored to indicate whether they are expressed primarily in apyrene sperm (gold), eupyrene sperm (purple), or unbiased between the morphs (gray).

Considering the quantitative results, patterns of differential abundance were asymmetrical between apyrene versus eupyrene sperm (**Figure 4**). Over 50 eupyrene-biased proteins showed > 8-fold enrichment, while only one apyrene-biased exceeded this same threshold. This asymmetry was also present in the qualitative differences, with 33 eupyrene-specific proteins identified versus eight apyrene-specific proteins. Considering both qualitative and quantitative assessments,125 proteins were apyrene-biased while 150 proteins were eupyrene-biased.

### 3.4 Functional analysis

Overall, approximately 85% of identified sperm proteins received a functional annotation from either PANNZER or BLAST, and 75% were annotated by both methods, which were generally consistent. However, the proportion of annotated proteins differed among the three subsets. For example, 80% of both apyrene-biased and unbiased proteins were annotated by PANNZER, but only 57% of eupyrene-biased proteins were annotated, which is a statistically significant difference in the rates of annotation (χ² = 41.24, df = 2, p <10^-8^). BLAST annotations followed a very similar pattern.

**Figure 5:**
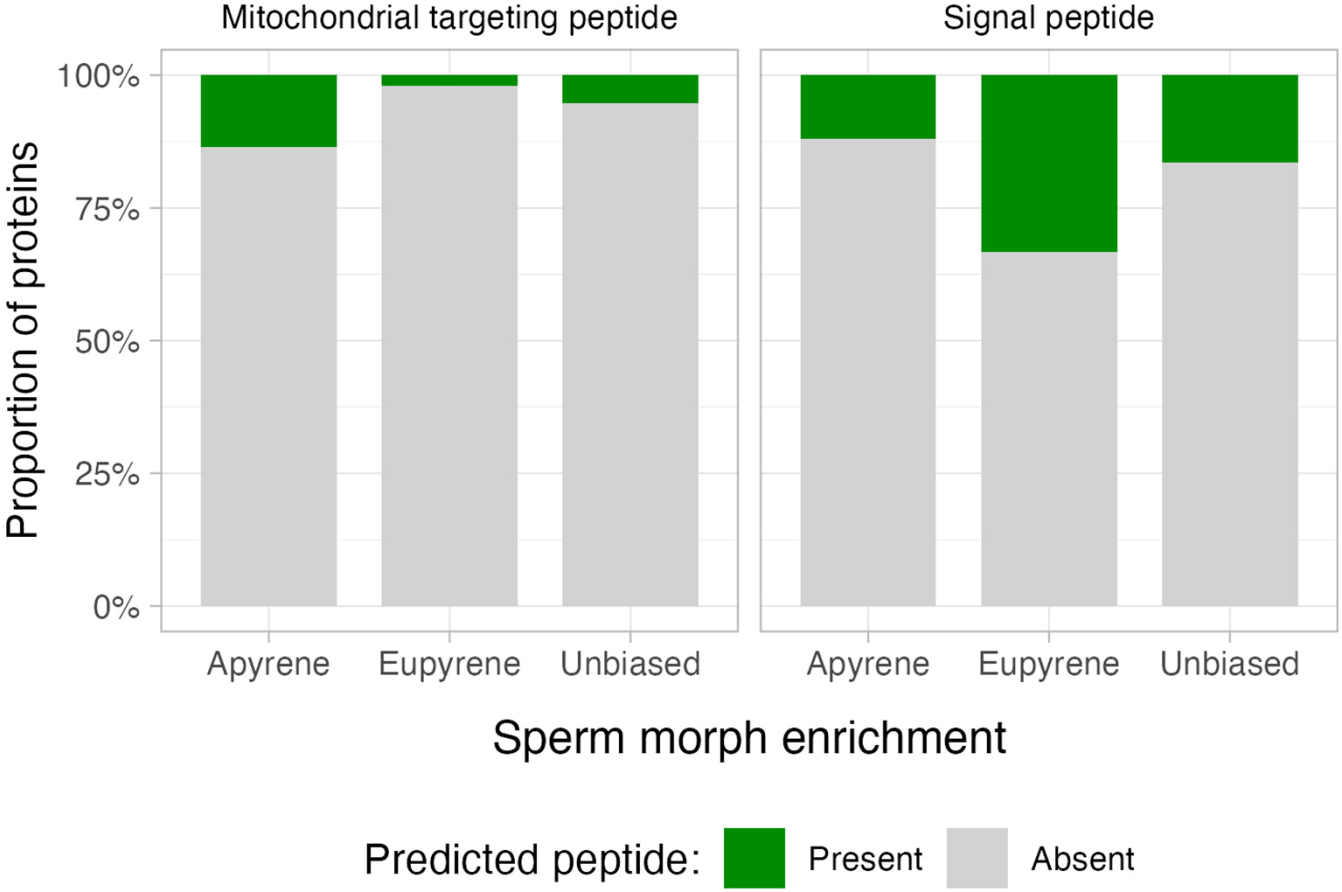
*Proportions of predicted targeting proteins among* sp*erm subsets.* The proportion of proteins annotated with a predicted mitochondrial targeting protein (left) or signal peptide (right) for apyrene-biased, eupyrene-biased, or unbiased subsets of the sperm proteome.

The three sperm subsets also differed in the frequencies of targeting signals (**Figure 5**). For mitochondrial targeting peptides, the proportions varied significantly (χ² = 18.8, df = 2, p <10^-4^), with apyrene-biased proteins showing notable enrichment relative to the other subsets. For signal peptides, proportions also varied significantly among sperm subsets (χ² = 29.6, df = 2, p <10^-6^). But in this case, it was eupyrene-biased proteins showing a notably higher frequency of predicted signal peptides. Finally, the frequency of predicted transmembrane domains also varied significantly between the three subsets (χ² = 13.2, df = 2, p <10^-2^). However, for transmembrane domains, the magnitude of differences between subsets was relatively modest, suggesting this result may not indicate important functional differentiation between sperm morphs.

Functional annotation results for each sperm protein are given in **Supplementary Table 1**. Counts and proportions of annotations for targeting peptides and transmembrane domains are given **Supplementary Table 2**.

### 3.5 Description of the sperm proteome composition

Proteins found to be biased towards the apyrene sperm had functions related to the formation of connective tissue, transportation into the mitochondrial inner membrane, and growth factors. Top eupyrene-biased proteins are often without functional annotations. Notably, the topmost biased protein is annotated by PANNZER as ISY1 protein, but it has no corresponding BLAST hit in SwissProt (**Supplementary Table 1**), suggesting this is a mis-annotation. In nearly all other cases, BLAST and PANNZER annotations are consistent. Among those top eupyrene-biased proteins, functions associated with malic acid metabolism and N-acetyltransferase enzymes occur repeatedly. Among proteins undifferentiated between morphs, many of the most abundant were also unannotated. Proteins found to be shared between the two sperm morphs were annotated as either seminal fluid proteins or proteins that are common components of sperm, such as tubulin.

### 3.6 Gene ontology

Gene Ontology term enrichment was assessed for the three GO categories: Biological Process (BP), Cellular Component (CC), and Molecular Function (MF). The three subsets of the sperm proteome were assayed for each GO category, yielding nine combinations of GO category and sperm-subset. For seven of these groupings, between nine and 20 significantly enriched terms were identified. The two exceptions were MF in the shared subset, with only three significant terms, and BP for apyrene-biased proteins, which yielded 32 significantly enriched terms. Notably, a third of these 32 BP terms for apyrene-biased proteins reference mitochondrial membrane activity or respiration, including the tricarboxylic acid cycle. Another noteworthy term indicates regulation of the ERK1/2 cascade, an intracellular signaling pathway implicated in several aspects of sperm function (40). Enriched BP terms for eupyrene-biased proteins often reflected transmembrane transport activity (e.g., proton, inorganic ion, cation, and anion). Other functions include pH reduction and vacuolar acidification. The shared proteome contained proteins enriched for terms associated with metabolic processes (e.g., peptide, cellular macromolecule, organic substance, cellular nitrogen compound, regulation of cellular processes and macromolecule metabolic processes) in addition to oogenesis and gamete generation.

For CC terms, over half (7/13) of the terms enriched among apyrene-biased proteins were mitochondria-related, a pattern similar to the BP result. However, the genes underlying this abundance of mitochondrial terms were notably distinct between the CC and BP categories. Of the 12 genes for BP and 18 for CC, only eight genes overlapped. Collectively, these 22 apyrene-biased genes represent a range of mitochondrial functions, including key components of oxidative metabolic processes (e.g., PIERAPG00000006982, a cytochrome c oxidase subunit), structural elements (e.g., PIERAPG00000004212, a prohibitin), and also chaperone proteins (e.g. PIERAPG00000002927, a heat shock protein). For the eupyrene-biased proteins, enriched CC terms predominately reflected membrane structures and transport, particularly including V-type ATPase functions. The shared proteome exhibits proteins enriched for terms relating to protein processing (e.g. ribosomal complex; Golgi apparatus), and a few other broad cytosolic functions.

For MF terms enriched among apyrene-biased sperm proteins, a few clearly relate to mitochondrial respiration (e.g. isocitrate dehydrogenase (NAD+) activity; oxidoreductase activity), but many terms indicate a range of other enzymatic and binding functions. Enriched MF terms for eupyrene-biased proteins strongly reflect transmembrane transporter activity. Only three enriched MF terms were reported for the shared proteome, all apparently related to protein translation (e.g., RNA and protein binding).

Complete results for GO term enrichment tests are provided in **Supplementary Table 3**. The 22 apyrene-biased proteins associated with enriched BP and CC mitochondria-related terms are provided in **Supplementary Table 4**.

### 3.7 Comparative homology analysis

**Figure 6:**
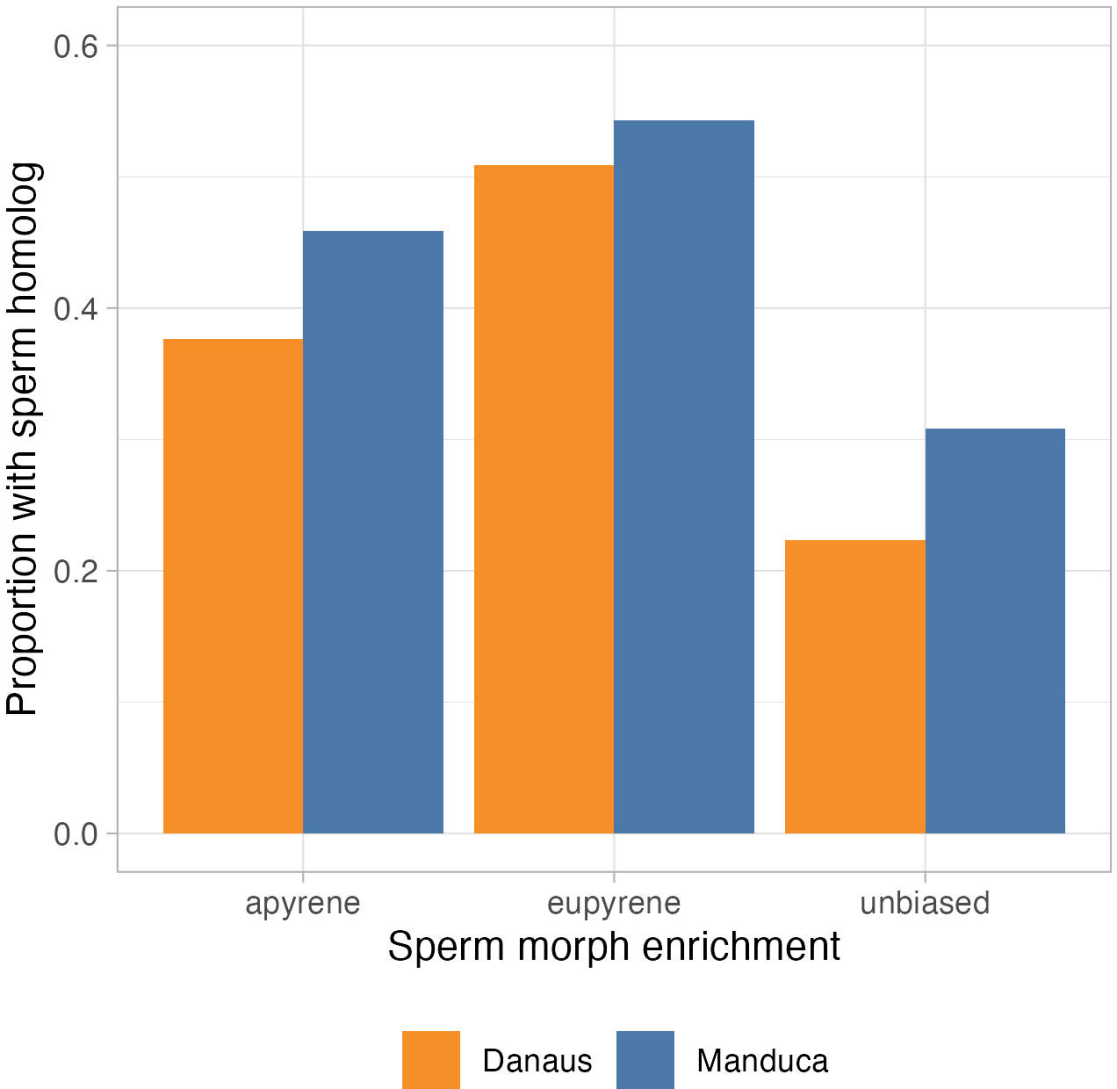
*Sperm protein homology*. Proportions of *Pieris rapae* sperm proteins with orthologs found in the sperm proteome of either *Danaus plexippus* (orange) or *Manduca sexta* (blue).

Comparisons between the *M. sexta* sperm proteome and the *P. rapae* sperm proteome reveal a higher count of sperm proteins in common than *D. plexippus* and *P. rapae* despite the latter two species being more closely related (**Figure 6**). The number of sperm homologs is statistically significantly different between the three sperm proteomes in both *D. plexippus* and *M. sexta* (p < 10^–5^ for both comparisons via chi-square test). In both comparisons, the eupyrene-biased proteins are more frequently shared between the species within the sperm samples, though a majority of unbiased proteins (those found in both the apyrene and eupyrene sperm) remain unique to each species.

## 4 Discussion

Using MS proteomics, we have identified more than 1,600 proteins from sperm in *Pieris rapae.* Overall, the number of proteins identified in the *P. rapae* sperm samples represents roughly twice as many proteins as compared to previous studies on Lepidopteran sperm proteomes (19). For instance, similar proteomic studies on *M. sexta* and *D. plexippus* have identified around 500–700 total sperm proteins (19, 20). One explanation for this greater number of proteins identified could be due to true biological differences: there could well be more proteins present in the *Pieris* sperm proteome, perhaps reflecting differences in remating rates. For example, a study done by Bissoondath and Wiklund states that *P. rapae* is a highly polyandrous species and that protein content of ejaculates increases with polyandry (41). Although in this case, they are studying bulk quantity of protein in ejaculates, protein content may also be related to complexity and therefore there is the possibility of more sperm proteins. However, *D. plexippus* also remate quite frequently, with up to 12 spermatophores observed in one female (20, 42). Since *D. plexippus* is one of the species with many fewer observed sperm proteins, this undermines the idea that increased polyandry is associated with increased complexity among sperm proteins.

Alternatively, differences in the count of identified proteins between *P. rapae* and the previous studies may more likely reflect methodological differences. Specifically, the previous studies used in-gel tryptic digests of isolated proteins prior to MS analysis (19). In-gel digests are well known to be less sensitive and recover fewer of the original sample’s proteins than the in-solution digests used in this study (43). Further, the difference in the number of identified proteins could be due to the sensitivity of the mass spectrometers themselves. Much like with advances in DNA sequencing, MS machine sensitivity and accuracy have improved notably in the nearly ten years since the previous proteomic studies on Lepidopterans were completed (44, 45). Considered broadly, the numbers of sperm proteins obtained from Lepidoptera are in the same general range reported from the few other similar studies on insects, but which have also been variable in terms of the total number of identified sperm proteins (46–48).

A notable asymmetry occurred in the number and magnitude of differentially abundant proteins, with more and stronger bias eupyrene-biased proteins. This presumably reflects major differences in the presence of organelles and other structures between the sperm morphs: eupyrene sperm have a nucleus, nuclear DNA, and endoplasmic reticulum while apyrene sperm do not. Thus, it is not surprising that we find this excess among eupyrene-biased proteins within our *P. rapae* samples. This pattern is consistent with previous studies that, while not quantitative, did report detecting notably more eupyrene than apyrene proteins (19, 27).

Due to these differences in cellular structures, and that eupyrene sperm are hypothesized to participate in fertilization, we expected that eupyrene sperm would have protein functions enriched for membrane transport and secretion, DNA structure and binding, and protein modification/binding activity. We expected that apyrene sperm would not have functions related to those of eupyrene-biased proteins and that they would have functions related to one of the three previously mentioned hypotheses: (1) to mediate sperm competition, (2) to aid in eupyrene sperm fertilization, and (3) to provide nuptial gifts and nutrients (12). Also, the structure of the nebenkern (sperm mitochondrial derivatives) differs between the sperm morphs (49).

Considering these expectations, gene ontology analyses revealed two important findings: (1) eupyrene GO terms were associated with pH regulation, ion transportation, V-type ATPase, and vacuolar acidification and (2) apyrene GO terms were broadly enriched for mitochondrial function and cellular respiration. For the first observation, eupyrene sperm, but not apyrene, have a vacuole-like structure that resembles the acrosome found in mammalian sperm. In mammalian sperm, the acrosome is a modified vacuole containing secreted proteins that allow the sperm to fuse with the egg through reactions mediating interactions between the membranes of the two cells (50). In mammals, V-type ATPase are necessary for acrosome acidification, which activates proteases stored in the acrosome (51). Many insect sperm contain highly similar and presumably analogous organelles; however, they do not necessarily function in the same way as a mammalian acrosome (52). The excess of proteins with signal peptides among eupyrene-biased proteins is consistent with this observation from GO terms, since such proteins will be destined for extracellular secretion (53, 54).

For the second observation, the apyrene-biased enrichment for mitochondrial targeting peptides and GO terms involving mitochondria and cellular respiration is noteworthy considering the currently ambiguous role of the nebenkern in insect sperm physiology. Some authors advocate that the nebenkern generally does not contribute to energy metabolism and that insect sperm primarily rely on glycolytic ATP production, not oxidative phosphorylation (OXPHOS) (48, 55). Rather, the nebenkern, which typically runs the length of the sperm tail, is thought to play primarily a structural role, consistent with it containing a prominent “paracrystalline” structure formed substantially by sperm leucyl aminopeptidase (S-Lap) proteins (56). One of the apyrene-biased proteins is annotated as a leucylaminopeptiase (PIERAPG00000008100), contains a predicted mitochondrial targeting peptide, and is also a sperm protein in *D. plexippus* and *M. sexta*, strongly suggesting this is a widely conserved lepidopteran S-Lap. This result tends to support a more “structural” role for the apyrene nebenkern.

Nonetheless, there are reports of oxidative respiration in sperm from some insect taxa, or depending on context (e.g., in male reproductive tract versus in female sperm storage organ) (55, 57). The strong apyrene-bias of several proteins directly functioning in the tricarboxylic acid (TCA) cycle and OXPHOS raises the possibility that apyrene sperm possess enhanced capacity for respiratory ATP synthesis compared to eupyrene sperm. Two additional, though still somewhat limited, lines of evidence indicate this is plausible. First, in silkmoths, Friedlander and Gitay (49) noted ultrastructural differences between sperm morphs in the nebenkern cristae. Eupyrene cristae were “poorly defined” in transverse sections compared to apyrene cristae. Because cristae increase inner mitochondrial membrane surface area and house the primary OXPHOS machinery, reduced cristae organization in eupyrene sperm could reflect diminished OXPHOS capacity. Second, Osanai (58) performed biochemical analysis of respiratory metabolism on the contents of silkmoth spermatophores, which contain both sperm morphs. He concluded, “glycolysis and the TCA-cycle function actively in the spermatophore, probably mainly in the apyrene and eupyrene sperm,” though no distinction was made between morphs in respirational capacity. These observations, combined with our proteomic results, highlight the intriguing possibility that Lepidoptera have partitioned roles of energy production between the two sperm morphs, such that apyrene sperm have greater capacity for ATP production via OXPHOS than eupyrene sperm.

Perhaps the most surprising result comes from the comparison of sperm proteomes between species of Lepidoptera. *P. rapae* and *D. plexippus* are both butterflies (superfamily Papilionoidea) and more closely related evolutionarily than either is to the moth *M. sexta* (superfamily Bombycoidea). Despite this, *P. rapae* and *M. sexta* share a higher proportion of homologous sperm proteins than the two butterfly species do. This could perhaps be an artifact of sampling and methodology or could represent a larger biological pattern. For example, *D. plexippus* may experience more rapid evolution and protein turnover than other lepidopteran species. Further sampling of additional species would confirm such a pattern or provide insights into an unexplored explanation.

In conclusion, the *P. rapae* sperm proteome data we collected will stand as a significant contribution towards research on sperm function, diversity, and evolution. Our results reveal molecular differences between apyrene and eupyrene sperm that correspond to known morphological differences, helping to bridge the gap between genotype and phenotype. The proteins identified within this study may offer specific targets for future applications, such as in pest management (Seth et al., 2023). This study also provides a rich foundation for future population genetic and molecular evolutionary studies that will elucidate the selective pressures experienced by dimorphic sperm. Finally, the sperm proteins we identified offer a rich set of targets for functional study, particularly in the context of male-female interactions in reproduction, which has a strong precedent in this species.

## Data Availability

The datasets presented in this study can be found in online repositories. The names of the repository/repositories and accession number(s) can be found below: PXD070767.

## Supplementary material

Supplementary Figure 1 *Images of* sp*erm samples.* Representative microscopy images of separated apyrene (left) and eupyrene (right) sperm fractions following panning. Note that each eupyrene bundle contains 256 individual sperm (12).

Supplementary Figure 2 *Overlap of protein detection in* sp*erm morphs.* Counts of proteins detected in mass-spectrometry analysis of separated *P. rapae* sperm morphs. Barplots show the frequency spectrum of protein detection (after filtering) across biological replicates for eupyrene and apyrene samples separately as well as all samples combined.

Supplementary Figure 3 *Patterns of missing protein observations across replicates.* Binary heatmaps show presence-absence patterns of protein detection across biological replicates from mass-spectrometry proteomic analysis of isolated sperm morphs from *P. rapae*. Proteins are classified as either missing at random (MAR; assumed to arise stochastically due to threshold detection effects) or missing not at random (MNAR; assumed to reflect systematic biological differences between sperm morphs); see methods for details. A missing value corresponds to the absence of detection for a given protein in a particular sample. A valid value indicates the protein was detected in a given sample.

Supplementary Table 1 *Comprehensive results (excel file)*. This table provides a comprehensive listing of results for sperm proteins identified from Pieris rapae via mass-spectrometry proteomic analysis. It includes identified proteins and abundance, differential abundance statistics, functional annotations, and orthology predictions.

Supplementary Table 2 *Targeting peptide annotations.* Counts and proportions of predicted targeting proteins or transmembrane domains for each of the three subsets of the Pieris rapae sperm proteome.

Supplementary Table 3 *Gene Ontology term enrichment results (excel file).* Gene Ontology (GO) term enrichment results are given for each GO category (Biological Process, Cellular Component, and Molecular Function) for proteins found to be apyrene-biased, eupyrene-biased, or unbiased from the differential abundance analysis.

Supplementary Table 4 *Apyrene mitochondrial GO genes (excel file).* Abridged functional annotations for the 22 proteins supporting enriched mitochondria-related Cellular Component and Biological Process Gene Ontology terms among apyrene-biased proteins.
