# Supplementary material for: Proteomic analysis of dimorphic sperm in the cabbage white butterfly, Pieris rapae
Source: Front Insect Sci. 2026 Mar 25;6:1772436. doi: 10.3389/finsc.2026.1772436 (PMC13060016; doi:10.3389/finsc.2026.1772436)
Supplement: Supplementary Figure 1 — Images of sperm samples. Representative microscopy images of separated apyrene (left) and eupyrene (right) sperm fractions following panning. Note that each eupyrene bundle contains 256 individual sperm (12). [file SupplementaryFile1.docx]

Supplementary Material

# Supplementary Figures and Tables

## Supplementary Figures


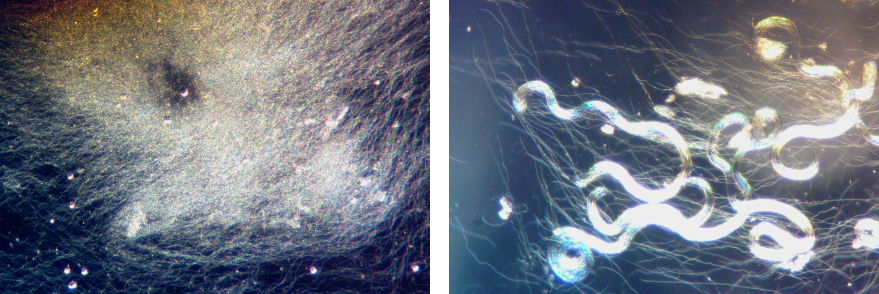


Supplemental Figure S1: Representative microscopy images of separated apyrene (left) and eupyrene (right) sperm fractions following panning. Note that each eupyrene bundle contains 256 individual sperm (Wedell and Cook, 1999).

***Supplemental Figure S2:*** ***Overlap of protein detection in sperm morphs.*** Counts of proteins detected in mass-spectrometry analysis of separated *P. rapae* sperm morphs. Barplots show the frequency spectrum of protein detection (after filtering) across biological replicates for eupyrene and apyrene samples separately as well as all samples combined.


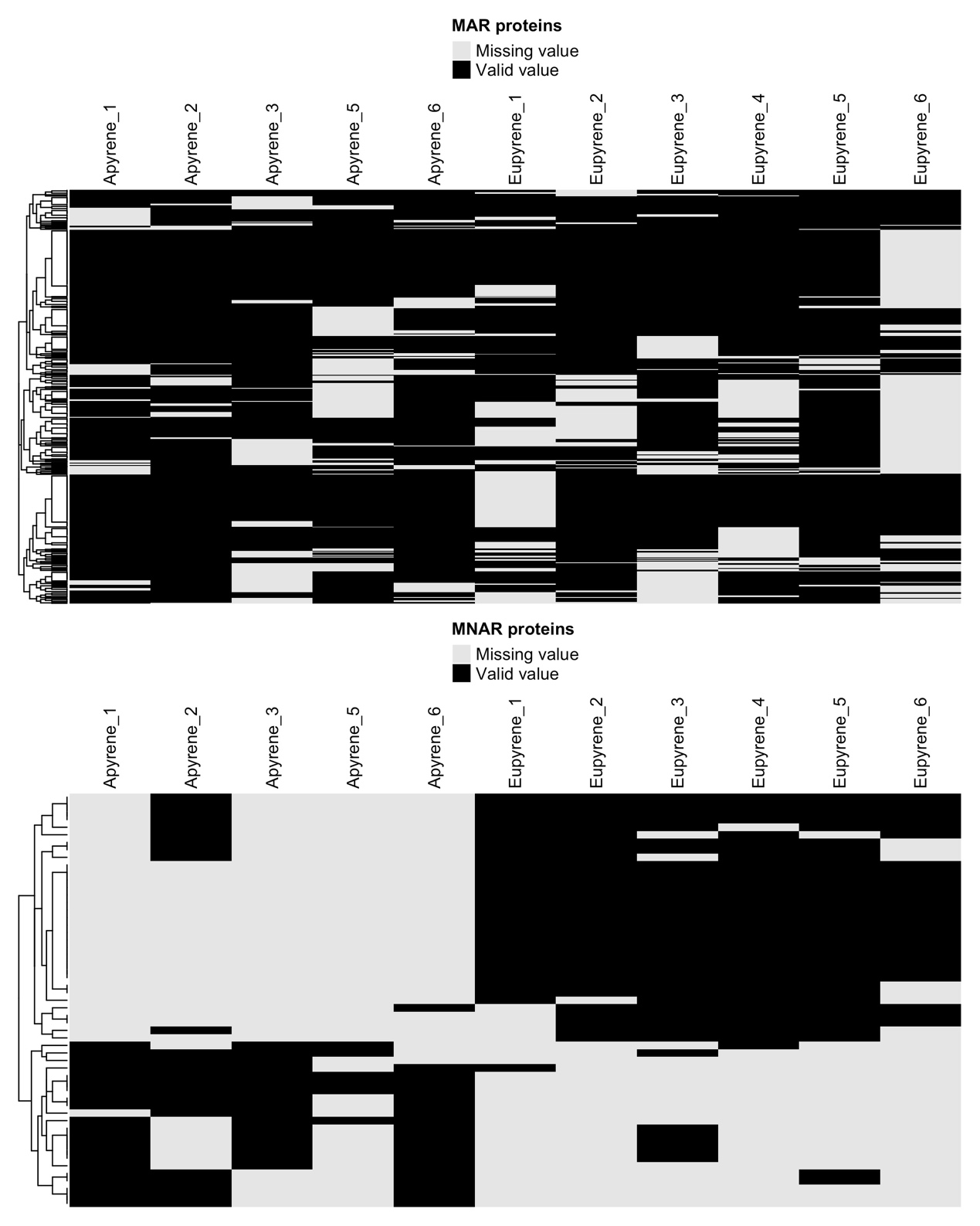


***Supplemental Figure S3: Patterns of missing protein observations across replicates.*** Binary heatmaps show presence-absence patterns of protein detection across biological replicates from mass-spectrometry proteomic analysis of isolated sperm morphs from *P. rapae*. Proteins are classified as either missing at random (MAR; assumed to arise stochastically due to threshold detection effects) or missing not at random (MNAR; assumed to reflect systematic biological differences between sperm morphs); see methods for details. A missing value corresponds to the absence of detection for a given protein in a particular sample. A valid value indicates the protein was detected in a given sample.

## Supplementary Tables

***Supplemental Table S2. Targeting peptide annotations.*** Counts and proportions of predicted targeting proteins or transmembrane domains for each of the three subsets of the Pieris rapae sperm proteome.

|  | Apyrene-biased | | Eupyrene-biased | | Shared | |
| --- | --- | --- | --- | --- | --- | --- |
|  | Count | Proportion | Count | Proportion | Count | Proportion |
| **Signal Peptide** |  |  |  |  |  |  |
| Present | 15 | 0.12 | 50 | 0.333 | 228 | 0.164 |
| Absent | 110 | 0.88 | 100 | 0.667 | 1159 | 0.836 |
| **Mitochondrial Targeting Peptide** |  |  |  |  |  |  |
| Present | 17 | 0.136 | 3 | 0.02 | 74 | 0.053 |
| Absent | 108 | 0.864 | 147 | 0.98 | 1313 | 0.947 |
| **Transmembrane Domain** |  |  |  |  |  |  |
| Present | 25 | 0.2 | 22 | 0.147 | 1247 | 0.899 |
| Absent | 100 | 0.8 | 128 | 0.853 | 140 | 0.101 |
